# Optical diagnosis in still images of colorectal polyps: comparison between expert endoscopists and PolyDeep, a Computer-Aided Diagnosis system

**DOI:** 10.3389/fonc.2024.1393815

**Published:** 2024-05-23

**Authors:** Pedro Davila-Piñón, Alba Nogueira-Rodríguez, Astrid Irene Díez-Martín, Laura Codesido, Jesús Herrero, Manuel Puga, Laura Rivas, Eloy Sánchez, Florentino Fdez-Riverola, Daniel Glez-Peña, Miguel Reboiro-Jato, Hugo López-Fernández, Joaquín Cubiella

**Affiliations:** ^1^ Research Group in Gastrointestinal Oncology Ourense, Hospital Universitario de Ourense, Ourense, Spain; ^2^ Fundación Pública Galega de Investigación Biomédica Galicia Sur, Complexo Hospitalario Universitario de Ourense, Sergas, Ourense, Spain; ^3^ Department of Computer Science, Escuela Superior de Ingenieria Informática (ESEI), CINBIO, University of Vigo, Ourense, Spain; ^4^ Next Generation Computer Systems Group (SING) Research Group, Galicia Sur Health Research Institute (IIS Galicia Sur), Ourense, Spain; ^5^ Department of Gastroenterology, Hospital Universitario de Ourense, Ourense, Spain; ^6^ Department of Gastroenterology, Hospital Universitario de Ourense, Centro de Investigación Biomédica en Red de Enfermedades Hepáticas y Digestivas (CIBEREHD), Ourense, Spain

**Keywords:** colorectal polyps, colonoscopy, deep learning, CADe/x, artificial intelligence, screening, convolutional neural networks

## Abstract

**Background:**

PolyDeep is a computer-aided detection and classification (CADe/x) system trained to detect and classify polyps. During colonoscopy, CADe/x systems help endoscopists to predict the histology of colonic lesions.

**Objective:**

To compare the diagnostic performance of PolyDeep and expert endoscopists for the optical diagnosis of colorectal polyps on still images.

**Methods:**

PolyDeep Image Classification (PIC) is an *in vitro* diagnostic test study. The PIC database contains NBI images of 491 colorectal polyps with histological diagnosis. We evaluated the diagnostic performance of PolyDeep and four expert endoscopists for neoplasia (adenoma, sessile serrated lesion, traditional serrated adenoma) and adenoma characterization and compared them with the McNemar test. Receiver operating characteristic curves were constructed to assess the overall discriminatory ability, comparing the area under the curve of endoscopists and PolyDeep with the chi- square homogeneity areas test.

**Results:**

The diagnostic performance of the endoscopists and PolyDeep in the characterization of neoplasia is similar in terms of sensitivity (PolyDeep: 89.05%; E1: 91.23%, p=0.5; E2: 96.11%, p<0.001; E3: 86.65%, p=0.3; E4: 91.26% p=0.3) and specificity (PolyDeep: 35.53%; E1: 33.80%, p=0.8; E2: 34.72%, p=1; E3: 39.24%, p=0.8; E4: 46.84%, p=0.2). The overall discriminative ability also showed no statistically significant differences (PolyDeep: 0.623; E1: 0.625, p=0.8; E2: 0.654, p=0.2; E3: 0.629, p=0.9; E4: 0.690, p=0.09). In the optical diagnosis of adenomatous polyps, we found that PolyDeep had a significantly higher sensitivity and a significantly lower specificity. The overall discriminative ability of adenomatous lesions by expert endoscopists is significantly higher than PolyDeep (PolyDeep: 0.582; E1: 0.685, p < 0.001; E2: 0.677, p < 0.0001; E3: 0.658, p < 0.01; E4: 0.694, p < 0.0001).

**Conclusion:**

PolyDeep and endoscopists have similar diagnostic performance in the optical diagnosis of neoplastic lesions. However, endoscopists have a better global discriminatory ability than PolyDeep in the optical diagnosis of adenomatous polyps.

## 1 Introduction

Colorectal cancer (CRC) is the third most common cancer worldwide and the second leading cause of cancer-related death (1, 2). Most CRCs develop from precursor lesions, adenomas, and serrated lesions, through a progressive transformation to carcinoma (1, 2). Population-based screening programmes are important for the detection and prevention of CRC and precancerous lesions in the average-risk population (50 to 75 years of age) (1–3). These programs are based on the use of immunochemical fecal occult blood tests as a preliminary screening method, and only those patients with positive results in this test are called for colonoscopy (1). Colonoscopy is the gold standard procedure for the detection of CRC, adenomas, and serrated lesions. Optical diagnosis aims to classify the colorectal polyps prior to resection (3, 4). However, due to the limited accuracy of the optical diagnosis performed by the endoscopists we still rely on the histological evaluation of resected lesions (5).

Artificial Intelligence is a discipline where systems are developed to perform tasks typically performed by humans. One of the primary areas in this research field is Machine Learning (ML), which encompasses Deep Learning (DL), a subarea that has garnered significant attention in recent years owing to the remarkable advancements achieved in both computer vision and natural language processing. DL was born as a specialization of neural networks, a family of ML models based on the connectionist principles of biological neural networks. DL models are characterized by their multilayered architecture and their particular connection patterns and activation functions, which allow them to effectively extract relevant features from unstructured data, such as images or natural language text. Consequently, DL has gained significant traction in the domain of medical image analysis, being now the basis of most Computer-Aided Diagnosis (CAD) systems recently developed (6–8). CAD system is a general term that encompasses the ability to detect and classify colonic lesions. There is a large amount of evidence of the impact of the Computer-Aided Detection (CADe) systems in diagnostic colonoscopy (9, 10). However, Computer-Aided Diagnosis (CADx) systems need more research and the information related to the optical diagnosis is, so far, limited with a wide area of improvement (8). In the available literature, there are reviews with meta-analysis and several controlled clinical studies of CADe and CADx (refers to the ability of detect and classify colorectal lesions respectively) systems which provide significant evidence of the benefits of integrating CAD systems in colonoscopies (8, 10). The implementation of these systems may improve and establish a minimum quality standard in clinical practice (10).

Given the clinical interest in the prevention of lower gastrointestinal disease and the increasing adoption of CADe/x systems, we decided to perform an *in vitro* analysis on still images of colorectal polyps to compare the optical diagnosis of expert endoscopists and PolyDeep, a CADe/x system developed by our research group in previous works (3, 9, 11, 12).

## 2 Materials and methods

### 2.1 PolyDeep Image Classification study design

The PolyDeep Image Classification (PIC) is a blinded, *in vitro*, diagnostic test study, aimed at comparing the optical diagnosis of endoscopists and PolyDeep, a CADe/x system. This study was approved by the Pontevedra-Ourense-Vigo Research Ethics Committee (2017/427).

### 2.2 PolyDeep development

PolyDeep is an artificial intelligence CADe/x system for the detection and characterization of colorectal polyps (3, 9, 11, 12). This system is composed of two DL models, capable of detecting and classifying polypoid lesions in real time during colonoscopy (11). A collection of colorectal polyp videos and images known as the Polyp Image BAnk database (PIBAdb) was used to train the PolyDeep models (13). This database is partially available as the PIBAdb Cohort through the biobank of the Galicia Sur Health Research Institute (13), although we expect to publish the full database in the near future. To build this database, the researchers obtained 709 videos (High-Definition White Light and Narrow Band Imaging-NBI) from 544 colonoscopies and 1603 polyps. Each video was reviewed and manually annotated by an expert to mark the main segments of interest, including those showing a polyp or an alteration such as the use of narrow band imaging (NBI) or the presence of an artefact (e.g. water, instrumental, etc.). From the polyp video segments, 44,477 still images with polyp and 14,124 without polyp were obtained (3, 9, 11). Polyp images were extracted (i) systematically, one image by second, focusing on detection model development, and (ii) manually, by experts endoscopists, looking for higher-quality still polyp images, focusing on the classification model development. All these extracted polyp images were labeled by expert endoscopists with a bounding box around the polyp location. Moreover, all polyps are associated with the endoscopic information (size, location, morphology, and predicted histology) and the final histological diagnosis as the gold standard. Colonoscopies were performed with endoscopes Olympus models 185 and 190 EVIS EXERA III CV-190 processors (Olympus, Tokyo, Japan) (3, 9).

### 2.3 Classification model development

The neural network architecture ResNet50 was used for the development of the colorectal polyp classification model (11). This model is integrated in the PolyDeep CAD and was used in the present work. It was pre-trained with the database ImageNet and only the last layer was fine-tuned with different datasets obtained from PIBAdb (11). The image sets used to develop the classification model were manually selected by expert endoscopists (11). PIBAdb images used for classification were divided into neoplasia (adenoma, sessile serrated lesions-SSL, traditional serrated adenomas-TSA) or non-neoplasia (hyperplastic). Other categories (non-epithelial neoplastic, invasive, and not histology) were discarded (11). The model was trained with 12933 selected NBI images from colonoscopies recorded between January 2018 and December 2022. While the volume of images may appear sufficient for model development, it is important to note that they are associated with only 827 polyps, resulting in relatively limited image diversity. In order to compensate this limitation, we performed a data augmentation strategy in the set of polyps used to train the classification model, by adding images from PIBAdb systematically extracted from polyp segments in the video (i.e., they have lower quality as they were not manually selected) (11). Using this strategy, we got 3436 lower quality NBI images and in each train partition of the cross-validation we increased on average 1666 images (11).

The classification model was developed using a 5-fold stratified cross-validation at polyp level, avoiding the inclusion of images from the same polyp in the training and validation partitions at the same time. This way, once the cross-validation was finished we obtained a confusion matrix to estimate the final performance of the classification model (11). With the images used in the 5-fold stratified cross-validation at polyp level only 491 NBI still images of colorectal polyps of the validation partition were included in PIC database. This gallery of images was classified by both PolyDeep and expert endoscopists. The classification model was developed using the Apache MxNet framework (https://mxnet.apache.org) with the GluonCV library (https://cv.gluon.ai), which provides DL models in computer vision. The dataset split into the training and validation partitions, as well as the training of each fold and results summarization, was performed using a Compi pipeline (11, 14).

### 2.4 Polyp Image Classification and image evaluation

We developed a custom tool for endoscopists to perform polyp classification called PIC (PolyDeep Image Classification) (**Supplementary Figure 1**). Using this tool, endoscopists classified 491 NBI still images (**Supplementary Figure 2**) showing only the content of the box used to delimit the polyp in the full image, as this is the same image used by the classification model to classify the polyps. These images have a mean width of 258.01 ± 107.96 pixels and a mean height of 249.20 ± 101.74 pixels. The average area of the images is 72756.95 ± 55719.99 pixels^2^. These images correspond to 491 polyps (69.04% adenomas, 14.87% SSL or TSA, and 16.09% hyperplastic), with a mean size of 6.43 ± 5.67 mm. All these boxed still NBI images, none of which included any landmark of the colon, were classified by the CADx and four expert endoscopists. The endoscopists participated in the CRC screening program with an adenoma detection rate in colonoscopy after a positive fecal immunochemical test ranging between 60 and 65%. The endoscopists classified polyps as neoplastic (either Adenoma, SSL, or TSA) or non-neoplastic (hyperplastic polyp), assigning a confidence level to their estimation (either high, medium, or low), while PolyDeep classified the lesions as neoplastic or non-neoplastic.

### 2.5 Statistical analysis

In the descriptive analysis, qualitative variables have been expressed as absolute frequencies and percentages, while quantitative variables have been expressed as means and standard deviations. The variables neoplasia and adenoma were the primary and secondary dependent variables, respectively. We evaluated the diagnostic accuracy of endoscopists and PolyDeep using 2x2 tables. We calculated sensitivity, specificity, Positive Predictive Value (PPV), Negative Predictive Value (NPV), Positive Likelihood Ratio (LR+), Negative Likelihood Ratio (LR-), Odds Ratio (OR), Youden Index (YI) and the F1-Score. Finally, we determined whether there were significative differences in sensitivity and specificity for neoplasia and adenoma characterization between endoscopists and PolyDeep using the McNemar test. Additionally, we used the Receiver Operating Characteristic curves (ROC curves) to calculate the Area Under the Curve (AUC) and compared them using the Chi-square homogeneity areas test. We used the statistical package R version 4.2.0 (The R Foundation for Statistical Computing, Institute for Statistics and Mathematics, Vienna, Austria) for the statistical analysis.

## 3 Results

### 3.1 Diagnostic performance of ResNet50 in optical diagnosis

The classification model, ResNet50, was trained with a set of images collected in PIBAdb. The diagnostic performance of the model was evaluated with a 5-fold stratified cross-validation at polyp level achieving a sensitivity of 84.76%, a specificity of 45.80%, and a Youden Index of 0.32.

### 3.2 Optical diagnosis of colorectal polyps

The flow chart in the (**Supplementary Figure 3**) shows the total number of colorectal polyp images that the endoscopists and PolyDeep evaluated, as well as the predicted histology. Two of the endoscopists classified all the images evaluated, while the others classified 88.80% and 95.28%. Finally, PolyDeep obtained the classification in 99.19% of the images evaluated. The number of neoplastic lesions (adenoma and serrated lesions) and non-neoplastic lesions (hyperplastic lesions) classified by the endoscopists and PolyDeep are shown in **Supplementary Table 1**. Endoscopists classified 83.95% (range 83.71%-84.27%) of the lesions as neoplastic lesions. Similarly, PolyDeep classified 84.39% of the lesions in the same category. In addition, this table summarizes the lesions that were correctly and incorrectly classified by the endoscopists and PolyDeep according to the histology. Endoscopists correctly classified 91.31% (range 86.65%-96.11%) of the neoplastic lesions. In this category, PolyDeep correctly classified 89.05% of the neoplastic lesions. On average, the endoscopists correctly classified 38.65% of the hyperplastic lesions (range 33.80%-46.83%) and PolyDeep 35.53% of the hyperplastic polyps (**Supplementary Table 1**). In **Supplementary Table 2** we show the number of colorectal lesions classified as adenomatous polyps (adenoma variable) or non-adenomatous polyps (SSA, TSA, and hyperplastic lesions). The endoscopists made a suitable classification in 84.97% (range: 65.78%-96.25%) of adenomatous polyps. In the same category, PolyDeep properly classified 90.24% of the lesions. The endoscopists and PolyDeep made an appropriate classification of non-adenomatous polyps in 50.69% (range: 39.13%-65.79%) and 26.17% respectively.

### 3.3 Evaluation of the diagnostic performance of endoscopists and PolyDeep

The diagnostic performance of the endoscopists and PolyDeep in optical diagnosis of neoplasia is shown in **Table 1**. We only found a statistically significant difference in sensitivity between one endoscopist (E2) and PolyDeep. There were no statistically significant differences in specificity. In **Table 2**, we show the diagnostic accuracy with respect to the optical diagnosis of adenoma. We observed an improved specificity of the endoscopists with respect to PolyDeep. On the other hand, only two endoscopists showed a statistically significant inferior sensitivity when compared with PolyDeep.

**Table 1:** Diagnostic accuracy of endoscopists and PolyDeep for neoplasia detection^1^.

	PolyDeep^2^	Endoscopists^2^							
		1	p^3^	2	p^3^	3	p^3^	4	p^3^
**Sensitivity**	89.05% (87.60-90.35)	91.23% (87.73-93.84)	0.51	96.11% (93.53-97.73)	<0.001	86.65% (82.89-89.71)	0.31	91.26% (88.00-93.73)	0.28
**Specificity**	35.53% (30.75-40.60)	33.80% (23.27-46.10)	0.84	34.72% (24.14-46.94)	1	39.24% (28.64-50.90)	0.86	46.84% (35.64-58.34)	0.22
**Positive Predictive Value**	88.19% (86.71-89.53)	87.63% (83.79-90.69)		88.76% (85.24-91.54)		88.15% (84.50-91.05)		89.95% (86.56-92.58)	
**Negative Predictive Value**	37.50% (32.52-42.75)	42.86% (29.97-56.73)		62.50% (45.81-76.83)		36.05% (26.17-47.18)		50.68% (38.82-62.48)	
**Positive Likelihood Ratio**	1.38 (1.28-1.49)	1.38 (1.16-1.63)		1.47 (1.24-1.74)		1.43 (1.19-1.71)		1.72 (1.39-2.12)	
**Negative Likelihood Ratio**	0.31 (0.26-0.37)	0.26 (0.16-0.41)		0.11 (0.06-0.20)		0.34 (0.24-0.49)		0.19 (0.13-0.28)	
**Odds Ratio**	4.48 (3.49-5.76)	5.31 (2.88-9.79)		13.15 (6.48-26.72)		4.19 (2.46-7.15)		9.20 (5.26-16.10)	
**Youden Index**	0.25 (0.20-0.30)	0.25 (0.14-0.36)		0.31 (0.20-0.42)		0.26 (0.15-0.37)		0.38 (0.27-0.49)	
**F1-Score**	0.88 (0.86-0.91)	0.89 (0.86-0.92)		0.92 (0.90-0.95)		0.87 (0.84-0.90)		0.90 (0.88-0.93)	

^1^Neoplastic lesion is defined as an adenoma, a sessile serrated lesion or a traditional serrated adenoma.

^2^Diagnostic accuracy of PolyDeep and each endoscopist is expressed in percentage with their confidence intervals.

^3^Results of the comparison between each endoscopist and PolyDeep using the McNemar chi-square test with continuity correction.

**Table 2:** Diagnostic accuracy of endoscopists and PolyDeep for adenoma detection^1^.

	PolyDeep^2^	Endoscopists^2^							
		1	p^3^	2	p^3^	3	p^3^	4	p^3^
**Sensitivity**	90.24% (88.70-91.59)	85.81% (81.25-89.43)	<0.01	96.25% (93.37-97.96)	0.07	65.78% (60.43-70.77)	<0.0001	92.04% (88.49-94.59)	1
**Specificity**	26.17% (23.08-29.52)	51.13% (42.35-59.84)	<0.01	39.13% (31.05-47.83)	<0.01	65.79% (57.60-73.16)	<0.0001	46.71% (38.64-54.95)	<0.01
**Positive Predictive Value**	73.49% (71.53-75.37)	80.00% (75.15-84.13)		78.57% (74.11-82.46)		81.09% (75.85-85.44)		79.39% (74.98-83.21)	
**Negative Predictive Value**	54.17% (48.86-59.38)	61.26% (51.51-70.22)		81.82% (70.01-89.86)		46.30% (39.54-53.18)		72.45% (62.35-80.76)	
**Positive Likelihood Ratio**	1.22 (1.17-1.28)	1.76 (1.47-2.10)		1.58 (1.38-1.81)		1.92 (1.52-2.43)		1.73 (1.48-2.01)	
**Negative Likelihood Ratio**	0.37 (0.31-0.45)	0.28 (0.20-0.38)		0.10 (0.05-0.17)		0.52 (0.43-0.63)		0.17 (0.11-0.25)	
**Odds Ratio**	3.28 (2.61-4.12)	6.33 (3.96-10.11)		16.50 (8.44-32.25)		3.70 (2.47-5.53)		10.13 (6.11-16.80)	
**Youden Index**	0.16 (0.13-0.20)	0.37 (0.28-0.46)		0.35 (0.27-0.43)		0.32 (0.22-0.41)		0.39 (0.30-0.47)	
**F1-score**	0.81 (0.77-0.84)	0.82 (0.79-0.86)		0.86 (0.83-0.89)		0.72 (0.68-0.76)		0.85 (0.82-0.88)	

^1^Adenoma is defined as adenomatous lesions and non-adenoma is defined as sessile serrated lesions, traditional serrated adenoma and hyperplastic lesions.

^2^Diagnostic accuracy of PolyDeep and endoscopists is expressed as percentage with their confidence intervals.

^3^Results of the comparison between each endoscopist and PolyDeep using the McNemar chi-square test with continuity correction.

### 3.4 Discriminative ability

**Figure 1:**
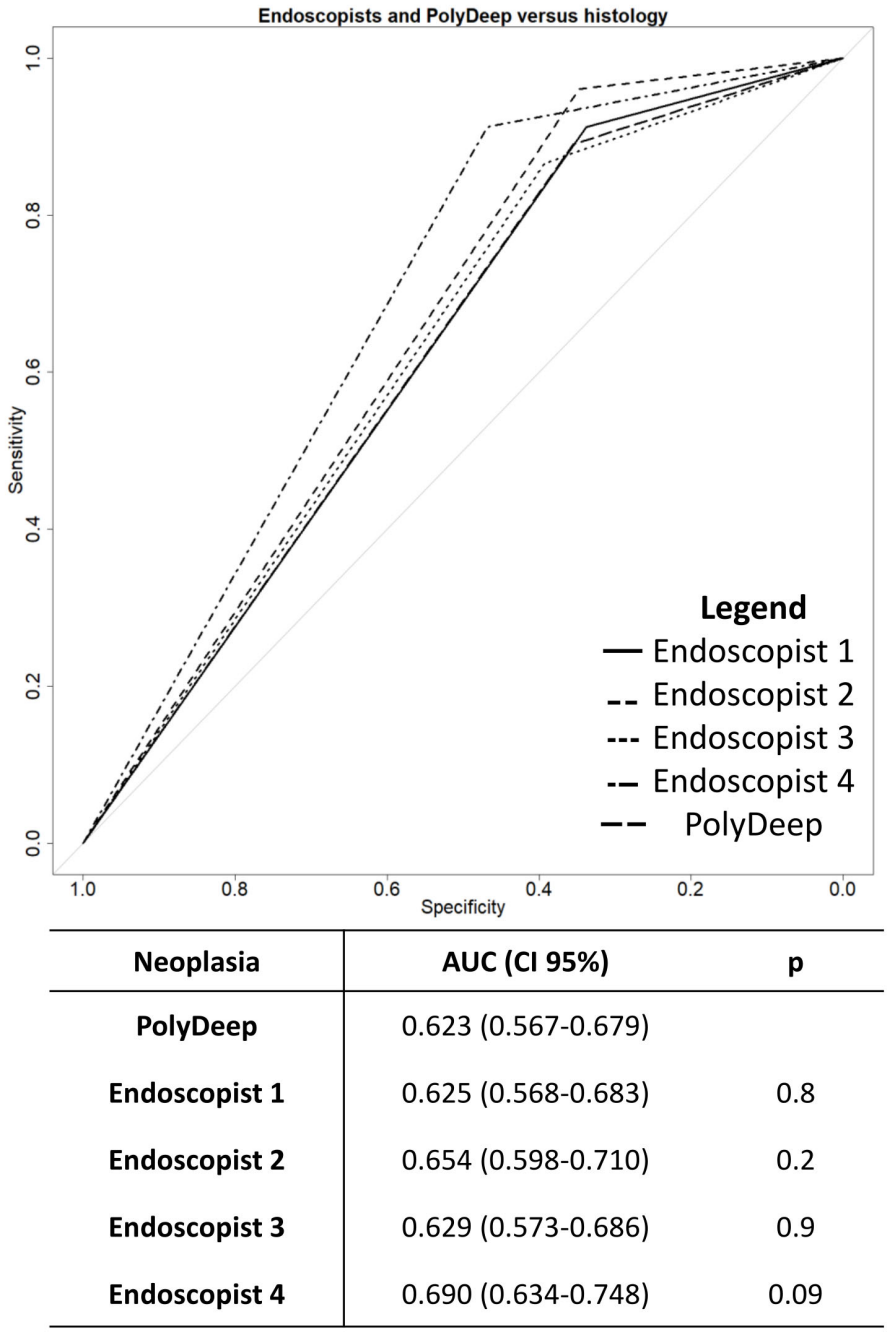
Receiver operating characteristics curves for neoplasia detection. AUC, Area Under the Curve; CI, Confidence interval; p, p-value.

**Figure 2:**
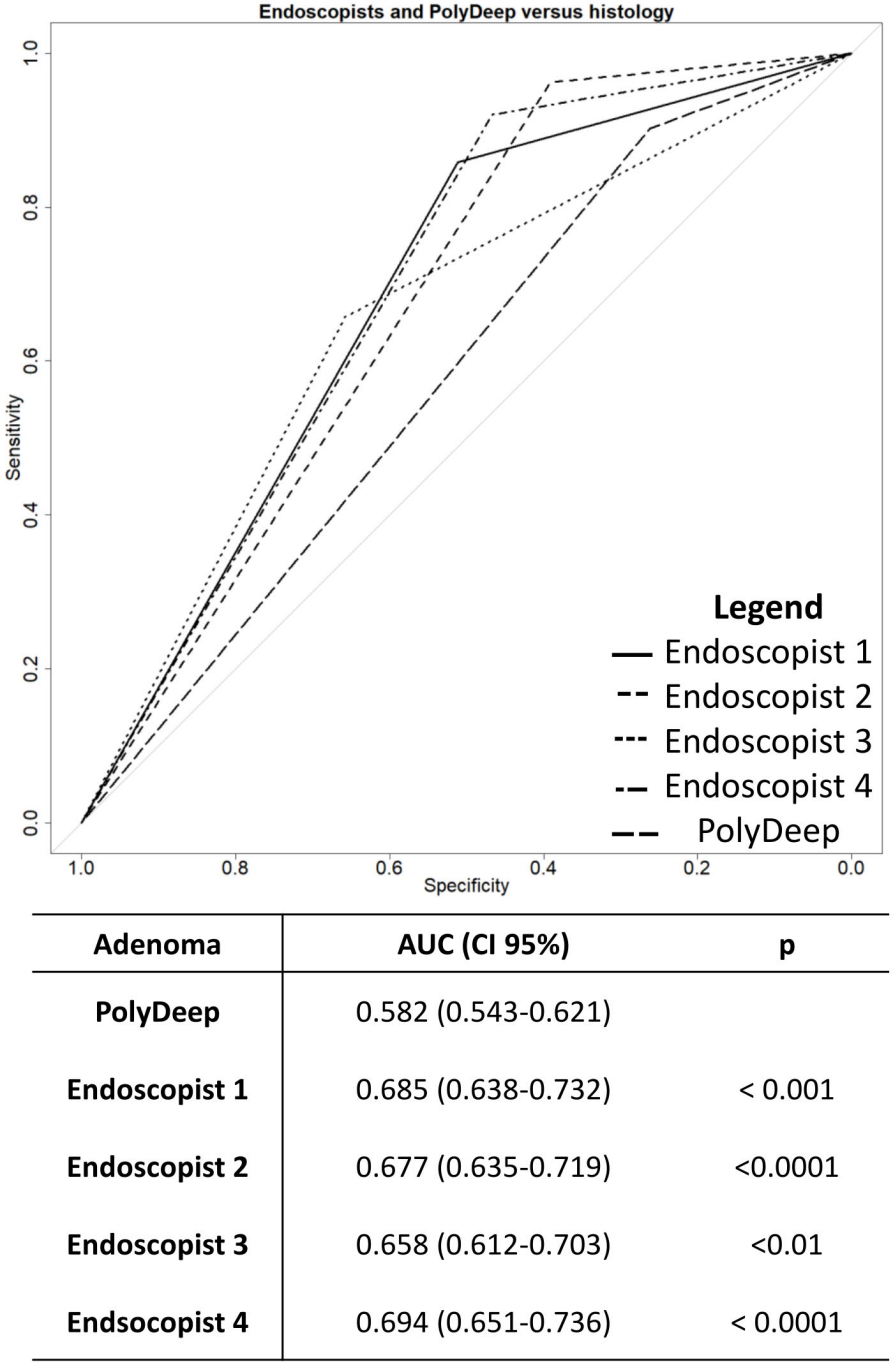
Receiver operating characteristics curves for adenoma detection. AUC, Area Under the Curve, CI, Confidence Interval; p, p-value.

The global discriminative ability of the endoscopists and PolyDeep was obtained performing ROC curves. In the overall discriminatory ability for neoplastic lesions, we did not detect any statistically significant differences between PolyDeep and expert endoscopists (**Figure 1**). In the ROC curves analysis for classification of adenoma (**Figure 2**) we detected statistically significant differences between PolyDeep and expert endoscopists.

## 4 Discussion

In our study we have evaluated and compared the diagnostic performance of PolyDeep and experienced endoscopists. We found that endoscopists had a similar diagnostic performance compared to PolyDeep for the characterization of neoplastic lesions. In contrast, PolyDeep was inferior to endoscopists for the correct diagnosis of adenomas. In **Supplementary Table 2** there are differences in the distribution of lesions between the categories adenoma (only includes adenomatous lesions) and non-adenoma (includes serrated and hyperplastic lesions). Therefore, serrated lesions are considered in the category non-adenoma. This assumes that certain lesions considered as neoplasms in **Supplementary Table 1** will not be considered as such in **Supplementary Table 2**. It is important to note that PolyDeep was specifically designed to classify neoplastic lesions (i.e. neoplastic vs. non-neoplastic), therefore the diagnostic accuracy for adenoma characterization was inferior to the endoscopists, as expected.

Several research articles have been published recently comparing the diagnostic performance of CADe/x systems with experienced and non-experienced endoscopists. These studies could be *in vitro* with imaging analysis or *in vivo* during a real colonoscopy procedure (15–23). The COACH study compared the diagnostic accuracy of a CADx system with two expert endoscopists in characterizing colorectal polyp images. As in our study, they differentiated between neoplastic and non-neoplastic lesions with a diagnostic accuracy of 78% (CADx), 84% and 77% (2 expert endoscopists) with no statistically significant differences. This CADx system obtained a better diagnostic performance than PolyDeep (sensitivity: 92.3% vs. 89.0%; specificity: 62.5% vs. 35.5%) (15). Our study could not compare expert and non-expert endoscopists with PolyDeep.

Van der Zander et al. (22) divided the lesions in the same categories as our study and classified 60 colorectal polyps using a pair of images in high-definition white light and blue light imaging for each polyp. The CADx system had a higher sensitivity (95.6%) than expert (61.1%) and non-expert endoscopists (55.4%). However, expert endoscopists (95.6%) had a superior specificity than CADx (93.3%) and less experienced endoscopists (93.2%). Finally, our study did not exclusively evaluate the diagnostic performance of endoscopists and PolyDeep in optical diagnosis of small polyps. Although we use images of diminutive or medium size polyps, we did not specifically evaluate the diagnostic performance in diminutive polyps (1-5 mm).

The POLAR system evaluated small polyps in a multicenter clinical validation setting, comparing the diagnostic ability between screening endoscopists with the CADx in the characterization of small colorectal polyps (1-5 mm) (18). The authors did not find any differences between the endoscopists and the CADx system evaluated. Furthermore, the CADx performance was similar to PolyDeep (sensitivity: 89.4% vs 89.0%; specificity: 37.8% vs 35.5%) (18).

The CADx systems of the COACH study and the POLAR study have not been evaluated with the same dataset as that of PolyDeep. However, comparing the diagnostic performance obtained by these two systems with PolyDeep, there are differences in their performances.

The dataset used in our study to evaluate the performance of PolyDeep and the endoscopists is imbalanced. Our data show a higher number of neoplastic lesions than non-neoplastic lesions. This is consistent with the normally distribution of lesions detected in a real clinical setting, where is more probable detect a neoplasm than a non-neoplasm. The F1-score and Youden Index show similar diagnostic performance between expert endoscopists and PolyDeep and are similar as we addressed with sensitivity.

During the development of the classification model, we observed that its performance was superior when NBI images were used. This observation is consistent with the findings of other studies, such as a meta-analysis by Lui et al. (24). Therefore, the images we used, both for model training and for endoscopist classification, were NBI images.

There are some *in vivo* studies that have evaluated the diagnostic performance of endoscopists and CADx systems (16, 19, 21, 24–27). In summary, the discriminatory ability of expert endoscopists and CADx systems is, at least, similar, with statistically significant differences when compared with novice endoscopists. In this sense, training with CADx systems can improve their optical diagnosis skills (19). The cost-effectiveness implications of using CADe/x systems require more research. If CADx systems improve the diagnostic performance irrespective of the endoscopist´s skill, strategies such as “diagnose and leave” or “resect and discard” could be widely used (28, 29). The Preservation and Incorporation of Valuable endoscopic Innovations (PIVI) document established that a 90% or higher NPV is required to apply the “diagnose and leave” strategy (23, 30). Moreover, optical diagnosis must correctly predict the histological diagnosis in at least 90% of lesions. According to our data and the available literature the application of both strategies in a real clinical setting is far from being applied (19, 29).

Our study has a several strengths. There are few studies comparing *in vitro* diagnostic performance of CADx and endoscopists (15, 22, 31). First, during CAD development, the endoscopists identified the location of the polyps in the images and later classified the same polyps in the PIC platform. Second, PolyDeep classified images corresponding to the test fold, which is a real-scenario where unseen images are presented to the CADx system. Third, all the images were collected prospectively from diagnostic colonoscopies with different levels of quality, close to the real colonoscopy setting.

On the other hand, our study has some limitations. The endoscopists had to adjust their optical diagnosis if they could only evaluate one single image per polyp, which, moreover, was limited to the minimal bounding box covering the polyp. This limitation could lead to an underestimation of their diagnostic performance. Due to the difficulty of classifying these images (**Supplementary Figure 2**), some endoscopists did not classify all of them. As a result, this could increase their correct classification ratio, showing a better performance in optical diagnosis of colorectal polyps. The endoscopists that classified all the images, independently of their quality (i.e. single low-quality images), could show worse results. This could influence negatively in their performance. In fact, in the actual clinical practice, endoscopists make their optical diagnosis using real time high-definition video. Another constraint is that we have a limited number of images with a limited number of polyps to train the classification model. We applied data argumentation to increase the average number of images to train the classification model and improve the diagnostic performance. The endoscopists predicted the histology of the colorectal polyps of the images based on their experience and not using the NICE classification. Finally, our results need to be validated in prospective studies based on *in vivo* evaluation of polyps during colonoscopy.

PolyDeep will be evaluated in three clinical trials (NCT05514301, NCT05512793 and NCT05513261) to determine its ability to detect and characterize colorectal lesions in real time. This evaluation of the diagnostic performance of the CADe/x system aims to determine whether the results obtained *in vitro* are transferred to a real colonoscopy setting. If the endpoints of these studies are met, the use of PolyDeep in a real colonoscopy procedure could improve the quality of the technique and provide a better patient care.

To conclude, our results are consistent with the literature, showing that PolyDeep a CADe/x system is equally accurate as experienced endoscopists for the optical diagnosis of neoplastic polyps (adenoma, SSL, and TSA).

## Data availability statement

The raw data supporting the conclusions of this article will be made available by the authors, without undue reservation.

## Ethics statement

The studies involving humans were approved by the Pontevedra-Ourense-Vigo Research Ethics Committee with the code (2017/427). The studies were conducted in accordance with the local legislation and institutional requirements. The participants provided their written informed consent to participate in this study.

## Author contributions

PD-P: Writing – original draft, Writing – review & editing. AN-R: Writing – original draft, Writing – review & editing. AD-M: Writing – original draft, Writing – review & editing. LC: Writing – original draft, Writing – review & editing. JH: Writing – original draft, Writing – review & editing. MP: Writing – original draft, Writing – review & editing. LR: Writing – original draft, Writing – review & editing. ES: Writing – original draft, Writing – review & editing. FF-R: Writing – original draft, Writing – review & editing. DG-P: Writing – original draft, Writing – review & editing. MR-J: Writing – original draft, Writing – review & editing. HL-F: Writing – original draft, Writing – review & editing. JC: Writing – original draft, Writing – review & editing.

## Supplementary material

Supplementary Figure 1 Interface of the PIC platform used by the endoscopists to classify the 2455 images collected in the study database. Each endoscopist had 491 polyps for made optical diagnosis.

Supplementary Figure 2 Original images vs. images to classify in the PolyDeep Image Classification (PIC) platform. **(A-H)** images in the above line are the original images. The images in the line below **(A-H)** are the images classified in the PIC platform (**Supplementary Material**).

Supplementary Figure 3 Optical diagnosis of the polyp images.

Supplementary Table 1 Optical diagnosis according to the final histological diagnosis. **Neoplastic:** includes the categories adenoma, SSA and TSA; **non-neoplastic** includes the category hyperplastic; qualitative variables are expressed as absolute frequencies and percentage. **Yes:** the endoscopist or PolyDeep classified the lesion correctly, while **No:** the endoscopist or PolyDeep misclassified the colonic lesion.

Supplementary Table 2 Optical diagnosis according to the adenoma histology. **Adenoma:** Adenoma variable only include the category adenoma. **Non-adenoma:** includes traditional serrated adenoma, sessile serrated adenoma and hyperplastic lesions. The variables showed in the table are categorical; therefore, they are expressed as absolute frequencies and percentage. **Yes**: the endoscopist or PolyDeep classified the lesion correctly, while **No**: the endoscopist or PolyDeep misclassified the colonic lesion.
