# Supplementary figures and images for: Optical diagnosis in still images of colorectal polyps: comparison between expert endoscopists and PolyDeep, a Computer-Aided Diagnosis system
Source: Front Oncol. 2024 May 23;14:1393815. doi: 10.3389/fonc.2024.1393815 (PMC11153726; doi:10.3389/fonc.2024.1393815)

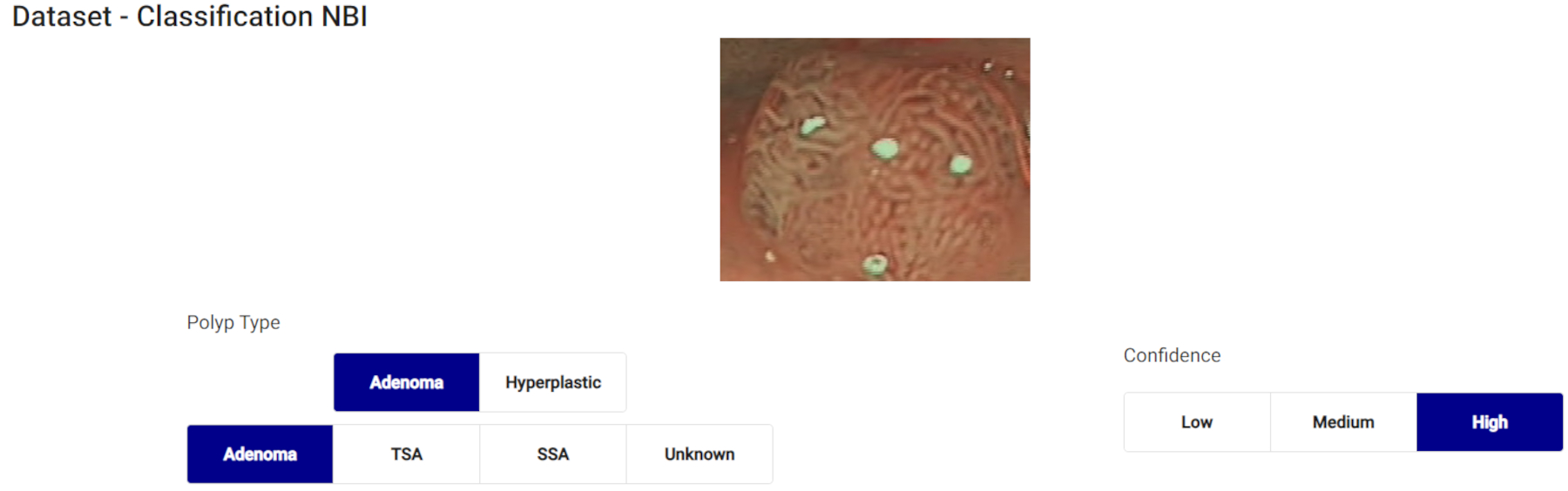

Supplement: Supplementary Figure 1 — Interface of the PIC platform used by the endoscopists to classify the 2455 images collected in the study database. Each endoscopist had 491 polyps for made optical diagnosis. [file Image_1.jpeg]

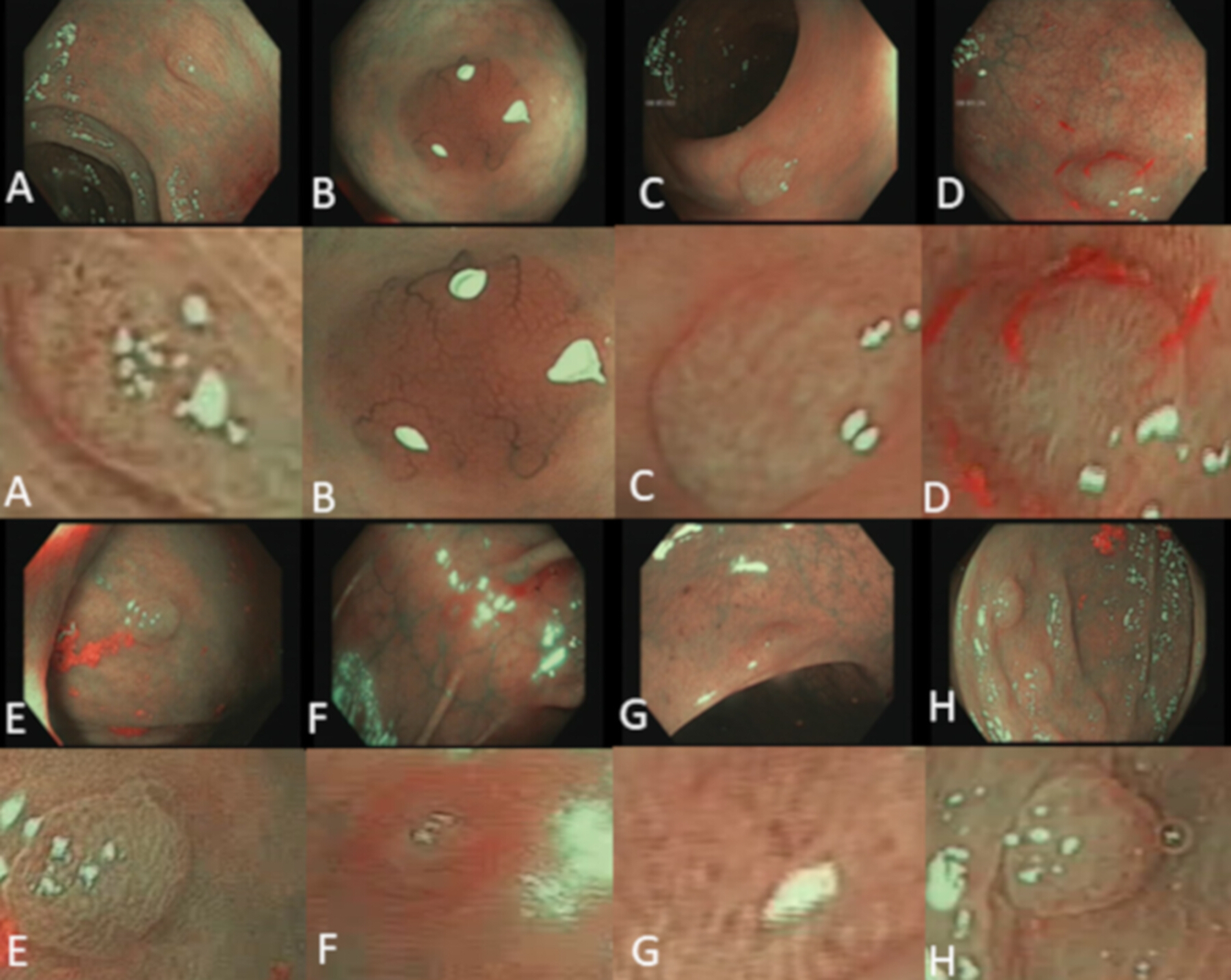

Supplement: Supplementary Figure 2 — Original images vs. images to classify in the PolyDeep Image Classification (PIC) platform. (A-H) images in the above line are the original images. The images in the line below (A-H) are the images classified in the PIC platform ( Supplementary Material ). [file Image_2.jpg]

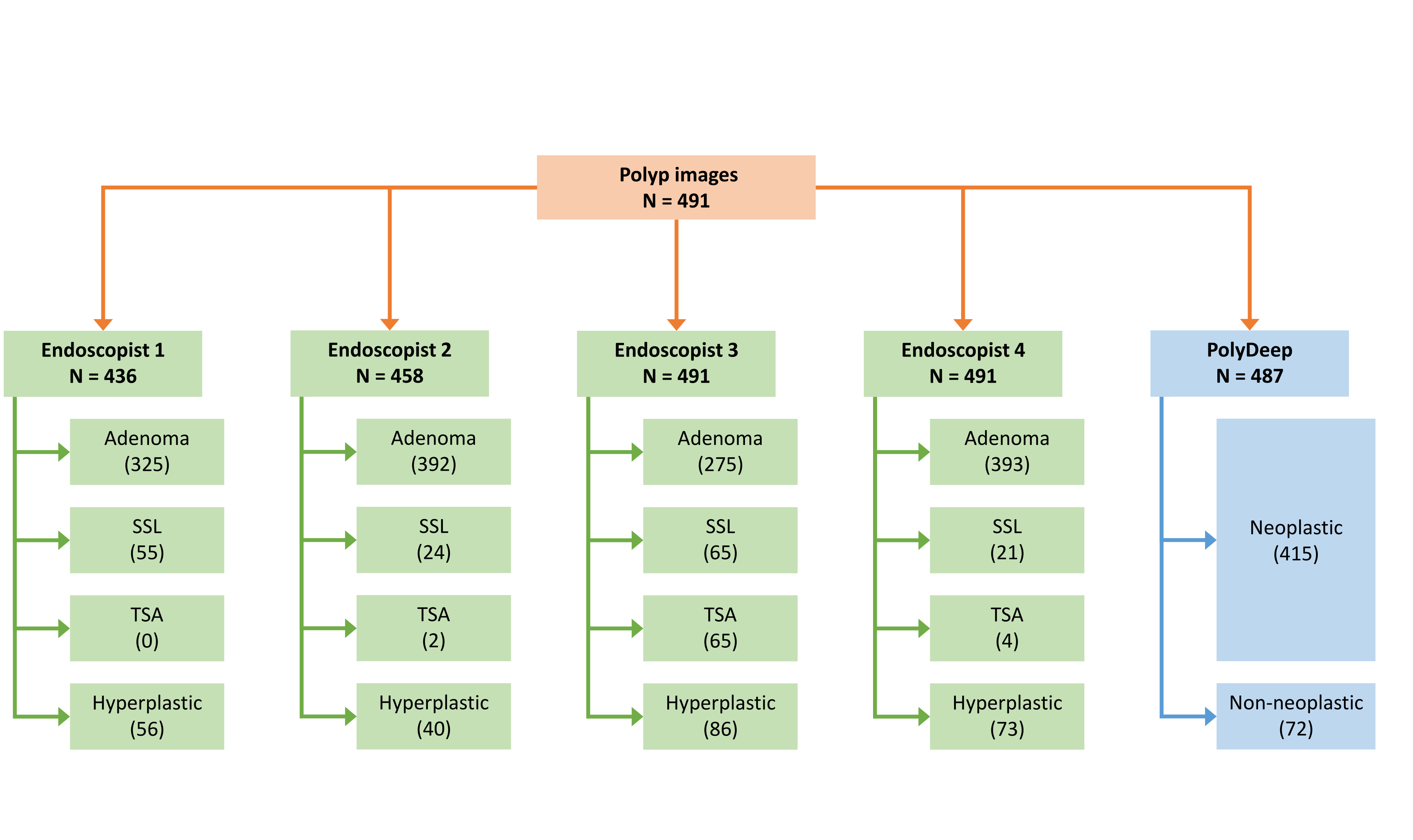

Supplement: Supplementary Figure 3 — Optical diagnosis of the polyp images. [file Image_3.jpeg]
