# Supplementary material for: Optical diagnosis in still images of colorectal polyps: comparison between expert endoscopists and PolyDeep, a Computer-Aided Diagnosis system
Source: Front Oncol. 2024 May 23;14:1393815. doi: 10.3389/fonc.2024.1393815 (PMC11153726; doi:10.3389/fonc.2024.1393815)
Supplement: Supplementary Table 1 — Optical diagnosis according to the final histological diagnosis. Neoplastic: includes the categories adenoma, SSA and TSA; non-neoplastic includes the category hyperplastic; qualitative variables are expressed as absolute frequencies and percentage. Yes: the endoscopist or PolyDeep classified the lesion correctly, while No: the endoscopist or PolyDeep misclassified the colonic lesion. [file Table_1.docx]

**Table 1 (supplementary material):** Optical diagnosis according to the final histological diagnosis

|  | **Histology** | | | |
| --- | --- | --- | --- | --- |
|  | **Neoplastic (n= 412)** | | **Non-Neoplastic (n=79)** | |
|  | **Yes n (%)** | **No n (%)** | **Yes n (%)** | **No n (%)** |
| **Endoscopist 1**  **N= 436** | 333  (80.83%) | 32  (7.77%) | 24  (30.37%) | 47  (59.49%) |
| **Endoscopist 2**  **N= 458** | 371  (90.04%) | 15  (3.64%) | 25  (31.65%) | 47  (59.49%) |
| **Endoscopist 3**  **N=491** | 357  (86.65%) | 55  (13.35%) | 31  (39.24%) | 48  (60.76%) |
| **Endoscopist 4**  **N=491** | 376  (91.26%) | 36  (8.74%) | 37  (46.84%) | 42  (53.16%) |
| **PolyDeep**  **N = 487** | 366  (88.83%) | 45  (10.92%) | 27  (34.18%) | 49  (62.03%) |

**Neoplastic:** includes the categories adenoma, SSA and TSA; **non-neoplastic** includes the category hyperplastic; qualitative variables are expressed as absolute frequencies and percentage. **Yes:** the endoscopist or PolyDeep classified the lesion correctly, while **No:** the endoscopist or PolyDeep misclassified the colonic lesion.
