# Supplementary material for: Optical diagnosis in still images of colorectal polyps: comparison between expert endoscopists and PolyDeep, a Computer-Aided Diagnosis system
Source: Front Oncol. 2024 May 23;14:1393815. doi: 10.3389/fonc.2024.1393815 (PMC11153726; doi:10.3389/fonc.2024.1393815)
Supplement: Supplementary Table 2 — Optical diagnosis according to the adenoma histology. Adenoma: Adenoma variable only include the category adenoma. Non-adenoma: includes traditional serrated adenoma, sessile serrated adenoma and hyperplastic lesions. The variables showed in the table are categorical; therefore, they are expressed as absolute frequencies and percentage. Yes: the endoscopist or PolyDeep classified the lesion correctly, while No: the endoscopist or PolyDeep misclassified the colonic lesion. [file Table_2.docx]

**Table 2 (supplementary material):** Optical diagnosis according to the adenoma histology

|  | **Histology** | | | |
| --- | --- | --- | --- | --- |
|  | **Adenoma (N = 339)** | | **Non-Adenoma (N= 152)** | |
|  | **Yes n (%)** | **No n (%)** | **Yes n (%)** | **No n (%)** |
| **Endoscopist 1**  **N= 436** | 260  (76.70%) | 43  (12.68%) | 68  (44.74%) | 65  (42.76%) |
| **Endoscopist 2**  **N= 458** | 308  (90.85%) | 12  (3.54%) | 54  (35.53%) | 84  (55.26%) |
| **Endoscopist 3**  **N=491** | 223  (65.78%) | 116  (34.22%) | 100  (65.79%) | 52  (34.21%) |
| **Endoscopist 4**  **N=491** | 312  (92.04%) | 27  (7.96 %) | 71  (46.71%) | 81  (53.29%) |
| **PolyDeep**  **N = 487** | 305  (89.97%) | 33  (9.73%) | 39  (25.66%) | 110  (72.37%) |

**Adenoma:** Adenoma variable only include the category adenoma. **Non-adenoma:** includes traditional serrated adenoma, sessile serrated adenoma and hyperplastic lesions. The variables showed in the table are categorical; therefore, they are expressed as absolute frequencies and percentage. **Yes**: the endoscopist or PolyDeep classified the lesion correctly, while **No**: the endoscopist or PolyDeep misclassified the colonic lesion. (supplementary material)
